# Genome-Scale Multilocus Microsatellite Typing of *Trypanosoma cruzi* Discrete Typing Unit I Reveals Phylogeographic Structure and Specific Genotypes Linked to Human Infection

**DOI:** 10.1371/journal.ppat.1000410

**Published:** 2009-05-01

**Authors:** Martin S. Llewellyn, Michael A. Miles, Hernan J. Carrasco, Michael D. Lewis, Matthew Yeo, Jorge Vargas, Faustino Torrico, Patricio Diosque, Vera Valente, Sebastiao A. Valente, Michael W. Gaunt

**Affiliations:** 1 London School of Hygiene and Tropical Medicine, London, United Kingdom; 2 Instituto de Medicina Tropical, Universidad Central de Venezuela, Los Chaguaramos, Caracas, Venezuela; 3 Centro Nacional de Enfermedades Tropicales, Santa Cruz, Bolivia; 4 Universidad Mayor de San Simon, Cochabamba, Bolivia; 5 Consejo Nacional de Investigaciones Científicas y Técnicas, Instituto de Patologia Experimental, Universidad Nacional de Salta, Salta, Argentina; 6 Instituto Evandro Chagas, Rodovia, Belem, Para, Brazil; The Pennsylvania State University, United States of America

## Abstract

*Trypanosoma cruzi* is the most important parasitic infection in Latin America and is also genetically highly diverse, with at least six discrete typing units (DTUs) reported: Tc I, IIa, IIb, IIc, IId, and IIe. However, the current six-genotype classification is likely to be a poor reflection of the total genetic diversity present in this undeniably ancient parasite. To determine whether epidemiologically important information is “hidden” at the sub-DTU level, we developed a 48-marker panel of polymorphic microsatellite loci to investigate population structure among 135 samples from across the geographic distribution of TcI. This DTU is the major cause of resurgent human disease in northern South America but also occurs in silvatic triatomine vectors and mammalian reservoir hosts throughout the continent. Based on a total dataset of 12,329 alleles, we demonstrate that silvatic TcI populations are extraordinarily genetically diverse, show spatial structuring on a continental scale, and have undergone recent biogeographic expansion into the southern United States of America. Conversely, the majority of human strains sampled are restricted to two distinct groups characterised by a considerable reduction in genetic diversity with respect to isolates from silvatic sources. In Venezuela, most human isolates showed little identity with known local silvatic strains, despite frequent invasion of the domestic setting by infected adult vectors. Multilocus linkage indices indicate predominantly clonal parasite propagation among all populations. However, excess homozygosity among silvatic strains and raised heterozygosity among domestic populations suggest that some level of genetic recombination cannot be ruled out. The epidemiological significance of these findings is discussed.

## Author Summary

The arrival of the *Trypanosoma cruzi* online genome now provides vital information for the study of Chagas disease. Using this resource, we identified and developed a genome-scale panel of rapidly evolving microsatellite markers that can be used to unravel the micro-epidemiology of this parasite. We then tested these against a panel of isolates belonging to the most widely occurring and ancient major lineage, *T. cruzi* I (TcI). Our study includes samples from across the geographical distribution of this lineage, including isolates from wild vectors, domestic vectors, as well as wild mammalian reservoirs and human hosts. This is the first time *T. cruzi* has been subjected to such high-resolution population genetic analysis. Our study shows that important epidemiological information lies at the intra-lineage level, especially when wild and domestic populations of parasite are compared. Crucially, in Venezuela, where Chagas disease may be resurgent despite decades of control effort, genotypes of parasites found in the wild are rarely represented in humans, despite evidence that infected wild vectors do invade houses. In this manuscript, we examine the epidemiological implications of this finding and others, and suggest how the approach we have developed can now be used to investigate the true nature of parasite transmission at Chagas disease foci throughout the Americas.

## Introduction

*T. cruzi*, the etiological agent of Chagas disease, is a vector borne zoonosis and considered the most important parasitic infection in Latin America. In excess of 10 million people are thought to carry the parasite, with ten times that number at risk (http://www.who.int). Consistent with a long history on the continent [1], *T. cruzi* ecology in the silvatic environment is highly complex. Over 73 mammalian genera and just over half of 137 described species of haematophagous triatomine bug are involved with parasite carriage and transmission [2],[3]. *T. cruzi* has an endemic range that stretches from the Southern USA to Northern Argentina. Most human infection is found in Central and South America and occurs primarily through contact with the contaminated faeces of domiciliated triatomine vector species.

Genotypic data support the existence of six stable discrete typing units (DTUs) in *T. cruzi*: TcI, TcIIa, TcIIb, TcIIc, TcIId, and TcIIe [4]. Greatest molecular divergence is observed between TcI and TcIIb [1],[4]. TcIIa and TcIIc have distinct genotypes but their affinities to other DTUs are inadequately understood [4],[5]. TcIId and TcIIe are hybrids, and have haplotypes shared across TcIIb and TcIIc [1],[6]. The ecological and epidemiological relevance of different *T. cruzi* DTUs have been the subject of considerable debate. Using a retrospective analysis of all available genotype records, we recently showed that diversification in the silvatic environment is likely to be driven by ecological niche as well as host species, with arboreal Didelphimorpha (opossums) the principal hosts of TcI, and terrestrial Cingulata (armadillos) the principal hosts of TcIIc [7]. TcI is a major agent for human disease north of the Amazon Basin [8],[9], but is also ubiquitous in silvatic transmission cycles throughout the Americas [10],[11]. In the Southern Cone region of South America, DTUs TcIIb, TcIId, and TcIIe cause most human infection [10]. With the exception of putative epizootic outbreaks [12], TcIIb, TcIId, and TcIIe are so far rare in the silvatic cycle [7].

The current six-genotype classification of *T. cruzi* is likely to provide a poor reflection of the total diversity present. Abundant evidence from nucleotide sequence [13],[14], microsatellite [5],[15], RAPD [16] and MLEE [11],[17] data exists to suggest that considerable genetic variation is hidden at the sub-DTU level. Combining an adequate sample size with a genetic marker of sufficient resolution to unravel fine-scale relationships, however, remains a significant challenge. Indeed few, if any, detailed studies exist to document the population genetic diversity of a mammalian protozoan parasite in its true silvatic cycle. For many zoonotic infections, e.g. *Cryptosporidium spp*, *Trypanosoma brucei sspp*, *Leishmania spp*, and *Toxoplasma gondii*, domestic mammals and (where applicable) associated vectors are the obvious target for population-level studies of parasite genetic variation since these are the most likely source of human outbreaks. For *T. cruzi*, this rationale must also extend to wild reservoir hosts. Many, especially opportunistic scavengers like *D. marsupialis*, also come into close contact with humans, either directly, or via infected silvatic vector species. In areas now free or without a history of vectorial domestic transmission, oral outbreaks are a growing concern [18].

High-resolution population genetic studies of other parasitic zoonoses have facilitated epidemiological tracking of human disease outbreaks, with obvious implications for the planning of effective disease control [19],[20]. Molecular methods transformed our early understanding of *T. cruzi* epidemiology, with the revelation that distinct transmission cycles (domestic/silvatic) could harbour different major lineages of parasite [21]. Predominantly clonal propagation observed in *T. cruzi* is in keeping with this result, where micro-endemic clones with characteristic host propensities, geographic distribution, medical significance and biological attributes should exist within the parasite population [22]. However, widespread multi-host *T. cruzi* lineages like TcI persist outside of this paradigm. With the advent of the *T. cruzi* genome [23], the stage is now set to re-examine the micro-epidemiology of human disease outbreaks in TcI in the context of ultra-high resolution genetic analysis and, crucially, silvatic parasite populations. In this study we have developed a multilocus microsatellite typing (MLMT) system for TcI and applied it to parasite isolates from throughout the Americas. While this is among the largest panel of isolates from a single DTU ever analysed, sample sizes are still restrictive. Similarly, widespread deviation from Mendelian sexuality in *T. cruzi* limits the inferences that can be made from standard population genetic analyses. To circumvent these issues, we largely avoided model-based population assignment protocols (e.g. Structure [24]). In spite of these limitations, we are able to identify key features of silvatic TcI populations and highlight population genetic processes that accompany a switch to the human host in two endemic areas. In doing so we show that the pattern of within-DTU parasite genetic diversity may contain vital epidemiological information in terms of control strategies, parasite pathogenesis and ultimately human disease.

## Results

A final dataset comprising 12,329 alleles (excluding missing data) from 135 isolates was subjected to analysis. Most strains presented one or two alleles at each locus. Multiple (≥3) alleles were observed at a small proportion of loci (0.98%) and only among strains not biologically cloned. Multiclonality, rather than aneuploidy, was determined to be the major source of this phenomenon by reference to analysis of a subset of nine microsatellite loci across 211 clones taken from a subset of eight strains that demonstrated multiple alleles at individual loci in the uncloned state (data not shown). Samples were allocated to seven populations: North and Central American (*AM*_North/Cen_), Venezuelan silvatic (*VEN*_silv_), North Eastern Brazil (*BRAZ*_North-East_), Northern Bolivia (*BOL*_North_), Northern Argentina (*ARG*_North_), Bolivian and Chilean Andes (*ANDES*_Bol/Chile_) and Venezuelan domestic (*VEN*_dom_). A full list of sample allocations is included in Table S2 and the rationale for the assignment of individuals to populations is detailed in the *Methods* section.

### Genetic diversity and rare allele frequency distributions

Greatest genetic diversity was observed in populations drawn from palm and lowland moist forest associated ecotopes in *VEN*_silv_, *BRAZ*_North-East_ and *BOL*_North_ (Allelic richness (A_r_) = 2.229–2.344, Table 1). Small, genetic-drift prone populations lose rare alleles at a faster rate than they can be replenished by mutation. Poisson-distributed rare allele frequency plots for *VEN*_silv_, *BRAZ*_North-East_ and *BOL*_North_ are, instead, characteristic of populations with a large, stable *N*_e_ at mutation-drift equilibrium (Figure S1) [25]. It is of note that patterns of both allelic richness and rare allele distribution are consistent across *VEN*_silv_ (n = 37) *BRAZ*_North-East_ (n = 39) and *BOL*_North_ (n = 16), largely independent of sample size (Table 1, Figure S1). Additionally, the size of geographic focus had little relevance in determining the amount of diversity present in these populations. A marginal reduction in allelic richness, for example, was observed between *BRAZ*_North-East_ and *BOL*_North_ (A_r_ = 2.344–2.229, Table 1), despite a massive reduction in sampling area (∼4,500,000 km^2^–10 km^2^).

**Table 1:** Population genetic parameters for seven TcI populations.

Population	N/G	MNA	A_r_ a	H_O_ b	H_E_ b	% HD^c^	%HE^d^	I_A_ e	P-Value^f^
*AM*_North/Cen_	7/7	1.92	1.532	0.332	0.445	0.00	0.00	2.39	0.005
*VEN*_silv_	37/37	6.45	2.337	0.449	0.637	44.19	0.00	1.38	<0.001
*BRAZ*_North-East_	39/39	6.67	2.344	0.383	0.571	50.00	0.00	2.03	<0.001
*BOL*_North_	16/16	4.67	2.229	0.467	0.643	17.50	0.00	3.98	<0.001
*ARG*_North_	10/10	2.41	1.794	0.535	0.551	8.82	2.94	12.37	<0.001
*ANDES*_Bol/Chile_	11/11	1.73	1.407	0.406	0.396	3.85	7.69	2.05	<0.001
*VEN*_dom_	13/13*	2.02	1.486	0.421	0.422	7.14	14.29	1.21	0.011

**Figure 1:**
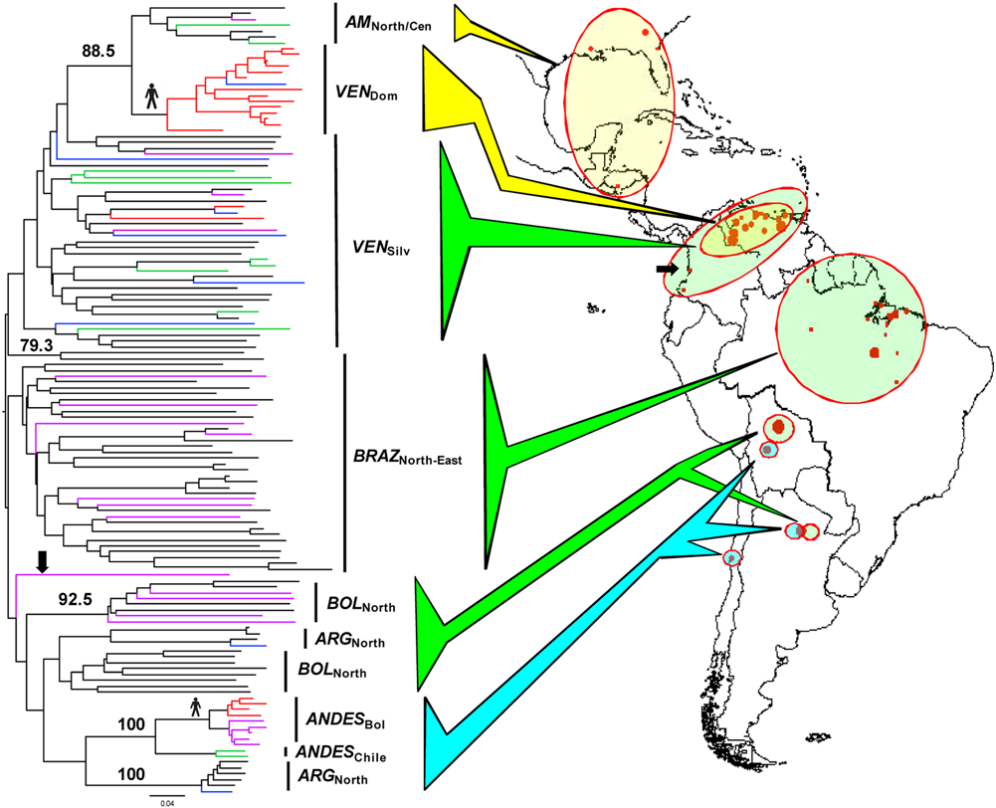
Unrooted neighbour-joining *D*_AS_ tree showing TcI population structure across the Americas. Based on the multilocus microsatellite profiles of 135 TcI isolates. *D*_AS_ values were calculated as the mean across 1,000 random diploid re-samplings of the dataset to accommodate multi-allelic loci. The presence of more than two alleles per locus did not disrupt the delineation of major clades (>90% majority consensus support). *D*_AS_-based bootstrap values were calculated over 10,000 trees from 100 re-sampled datasets, and those >75% are shown on major clades. Branch colour codes indicate strain origin. Black: *Didelphis* species; purple: non-*Didelphis* mammalian reservoir; green: silvatic triatomine; red: human; blue: domestic triatomine. Colored block arrows and circles indicate broad population types. Yellow: Venezuelan domestic and North/Central American groups; green: major silvatic populations; blue: South-Western clade. Black arrow indicates Colombian outlier assigned to Brazilian population. Human symbol indicates putative genetic association with domestic transmission. Closed red circle area is proportionate to sampling density. See text for details of population codes.

*: Two samples included in Figure 1 were excluded from population analysis due to DNA availability issues and consequential high levels of missing data.

a Allelic richness (sample size corrected).

b Mean observed and expected heterozygosity across all loci.

c Proportion of loci showing a significant deficit in heterozygosity after a sequential Bonferroni correction.

d Proportion of loci showing significant excess heterozygosity after a sequential Bonferroni correction.

e The Index of Association.

f P-value for index of association, calculated by comparison to a null distribution of 1,000 randomised datasets.

N, number of isolates in population; G, number of multilocus genotypes per population; MNA, mean number of alleles per locus.

A considerable reduction in diversity among silvatic isolates from *AM*_North/Cen_ was observed with respect to *VEN*_silv_, *BRAZ*_North-East_ and *BOL*_North_, again independent of sample size (A_r_ = 1.532, Table 1), concurrent with a reduction in rare allele frequency (Figure S1) and, assuming neutrality, implying that this population has been subject to a greater level of genetic drift in its recent past. Among three further populations, either exclusively comprised of domestic isolates (i.e. *VEN*_dom_), or including a mixture of domestic and silvatic isolates (i.e. *ANDES*_Bol/Chil_, *ARG*_North_), a reduction in diversity was also observed (A_r_ = 1.407–1.794). Here, to varying degrees, rare allele frequency plots again demonstrate a possible reduction in *N*_e_ by comparison to major silvatic populations (Figure S1).

### Heterozygosity indices

High levels of genetic diversity in the principal silvatic populations sampled (*VEN*_silv_, *BRAZ*_North-East_ and *BOL*_North_) gave rise to correspondingly large estimates of expected heterozygosity (*H*_E_ = 0.571–0.643, Table 1). However, observed levels of heterozygosity were substantially lower than those expected under Hardy-Weinberg Equilibrium (*H*_O_ = 0.383–0.467, Table 1) and statistical significance could be attached to this observation at the level of individual loci (Table 1). Silvatic isolates from *AM*_North/Cen_ demonstrated similar heterozygous deficit over loci, but, owing to sample size constraints, the same effect was not statistically supported at individual loci. In contrast to exclusively silvatic populations, observed levels of relative heterozygosity (*H*_O_∶*H*_E_, Table 1), were raised in populations that included domestic isolates, especially in *VEN*_dom_ (0.421∶0.422) and *ANDES*_Bol/Chile_ (0.406∶0.396).

To ascertain whether within-population subdivision had any effect on estimates of heterozygosity (i.e. Wahlund effects [26]), a number of subpopulations were picked (Table S2), representing, as far as possible, ‘true’ populations in space and time and within which no statistically supported genetic subdivision was observed on the basis of individual pair-wise distance measures (<75% bootstrap support, Figure 1). If a Wahlund effect was in operation, hidden population subdivision would act to artificially decrease observed heterozygosity levels (increase *F*_IS_). Mean *F*_IS_ estimates over loci across three silvatic populations, two from *BOL*_North_ and a further from *VEN*_silv_, instead remained positive (*F*_IS_ = 0.157) with a 99% confidence interval (CI) of 0.042∶0.288 obtained by bootstrapping over loci, thus providing non-probabilistic support for the deficit of heterozygosity as observed previously among the populations from which they were drawn, but also suggesting limited evidence of a Walhund effect. A similar analysis of *VEN*_dom_ and selected isolates from *ANDES*_Bol/Chile_ returned a negative *F*_IS_ value (*F*_IS_ = −0.157), although with a larger 99% CI encompassing zero (CI = −0.421∶0.12). A test for significant difference between *F*_IS_ values over loci between these sub-population groups (*BOL*_North_+*VEN*_silv_>*ANDES*_Bol_+*VEN*_dom_) generated by random shuffling of alleles between groups, was negative (p = 0.0639), albeit marginally, but suggests that direct comparisons of overall heterozygosity levels between these population groups should be approached with caution.

**Figure 2:**
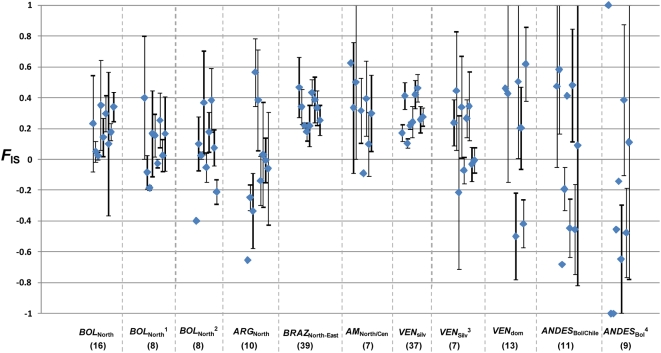
Mean *F*_IS_ values across loci on nine syntenous sequence fragments (SSFs) examined in eleven populations. Values suggest that gene conversion is a genomically diffuse process in homozygous silvatic populations. Error bars represent +/−standard error about the mean. Values without error bars correspond to SSFs containing only a single variable locus. Missing values correspond to SSFs containing no variable loci. Populations with postfix ^1,2,3,4^ are subsamples of larger populations. Numbers in parentheses indicate population size (n).

*F*_IS_ values were also analysed by syntenous sequence fragment (SSF) (as defined by the CL-Brener genome project; no chromosomal assembly is currently available), of which nine are represented in our panel with ≥2 microsatellite loci (Table S3, Figure 2). Calculations included both large and small (‘true’) population groupings for comparison. Mean *F*_IS_ values per SSF were consistently positive across major silvatic populations *BOL*_North_, *VEN*_silv_ & *BRAZ*_North-East_. This provides support for heterozygote deficiency at the population level, but also for a consistent level of heterozygosity between fragments. The same is broadly true for *AM*_North/Cen_, concomitant with an increase in error associated with both a reduction in genetic diversity and sample size. *F*_IS_ values for sub-population groupings from *BOL*_North_ (*BOL*_North_^1^ & *BOL*_North_^2^) and *VEN*_silv_ (*VEN*_silv_^3^) reflect those of their source populations. A marginal decrease in *F*_IS_ across some SSFs could be attributed to a Wahlund effect, and not uniquely to error, but major inconsistencies were not observed. In contrast, high inter-SSF variance was observed in both *ANDES*_Bol/Chil_ and *VEN*_dom_, and to a lesser extent *ARG*_North_, with some strongly negative values regardless of an increase in error about the mean. These data provide support for a distinction between these populations and those exclusively from the silvatic environment. At the sub-population level, the exclusion of Chilean isolates from *ANDES*_Bol_ did not have a major impact on the derived values, although error in this case was extremely high.

### Pair-wise measure of genetic distance

Figure 1 shows a Neighbor-joining tree based on pair-wise *D*_AS_ measures between individual isolates. Good bootstrap support was found for the grouping of isolates from *VEN*_dom_ and *AM*_North/Cen_ (88.5%), for subdivision within Argentinean isolates (100%), for subdivision within *BOL*_North_ (92.5%), as well as for the grouping of isolates obtained from the Bolivian and Chilean Andes. In the silvatic environment no clear diversification was observed by reservoir host, a phenomenon supported by a non-significant estimate of *F*_ST_ between *Didelphis sp.* and non-*Didelphis sp.* reservoir hosts in *BRAZ*_North-East_ (*F*_ST_ = 0.006, p = 0.594). Sample size restricts similar comparisons in other silvatic populations.

**Figure 3:**
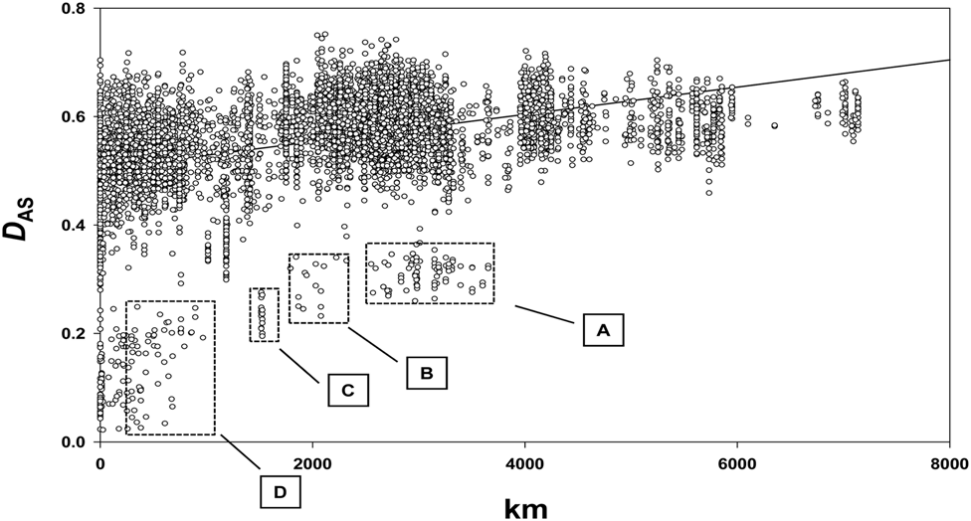
Continental scale spatial genetic structure among 135 TcI isolates from across the Americas. The graph shows a comparison between genetic (*D*_AS_) and geographic (km) distance across the entire dataset. Each data point represents a comparison between two isolates, and there are thus 9,180 in total. A significant positive correlation between these two measures was observed (R_XY_ = 0.394, p<0.0001). Outliers are highlighted by dashed lines. A – *VEN*_dom_ *vs AM*_North/Cen_; B – *AM*_North/Cen_ *vs AM*_North/Cen_; C − *ANDES*_Bol_ *vs ANDES*_Chile_, D – *VEN*_dom_ *vs VEN*_dom_. See text for details of population codes.

A portion of the pair-wise genetic diversification observed in the dataset could be attributed to isolation by distance (IBD). A Mantels test for matrix correspondence between pair-wise genetic (*D*_AS_) and geographic distance (km) revealed a highly significant positive correlation between these two measures (R_XY_ = 0.394, p<0.0001, Figure 3). Nonetheless, pair-wise comparisons also revealed considerable diversification between isolates from the same site in some instances (e.g. *BOL*_North_ - Mean *D*_AS_ = 0.479+/−0.009 (Standard Error)). Additionally, a number of outliers, representing comparisons within and between some groups of samples, are seen in Figure 3. These correspond to geographically disperse but relatively genetically homogeneous groups. Of particular interest are domestic isolates from Venezuela (*VEN*_dom_), comparisons between which lie within the dashed box labelled ‘D’ in Figure 3. No significant IBD is observed among these isolates when analysed separately (R_XY_ = 0.225, p = 0.0531) in contrast to those from the silvatic environment, which do show significant IBD (*VEN*_silv_ (Colombian outlier excluded, see Table S2) - R_XY_ = 0.292, p = 0.0001). A related observation is made among isolates from *AM*_North/Cen_, where no significant IBD (R_XY_ = 0.360, p = 0.161) is observed. Again, these isolates fall as outliers in Figure 3 (Box B).

### Population subdivision

Despite evidence of spatial structure across Amazonia at an individual level (Mantel's test *VEN*_silv_, *BRAZ*_North-East_, and *BOL*_North_ combined - R_XY_ = 0.533, p<0.0001) the level of subdivision between these populations was generally low (*F*_ST_ = 0.108–0.148, Table S1). Another observation not wholly consistent with IBD was a significant degree of subdivision between isolates from *ANDES*_Bol/Chil_ and *BOL*_North_ (*F*_ST_ = 0.304) as compared with the strong connectivity between *BOL*_North_ and more distant lowland populations (e.g. *BOL*_North_ - *VEN*_silv_ *F*_ST_ = 0.148, Table S1). Most striking was the high level of discontinuity implied by the *F*_ST_ estimate between populations *VEN*_dom_ and *VEN*_silv_ (*F*_ST_ = 0.295), which approximately overlap in their distribution. To place this observation in context, similar subdivision is seen between populations *VEN*_silv_ and *ARG*_North_ (*F*_ST_ = 0.226) which lie >5000 km apart.

### Linkage disequilibrium in TcI populations

Accounting for known physical linkage and excluding loci of unknown linkage group, the level of multilocus linkage disequilibrium was assessed using the I_A_, and was found to be statistically greater than a null distribution generated from 1000 random permutations in all populations (Table 1). Thus, the current dataset is consistent with predominant clonality in this parasite.

## Discussion

This study represents the most comprehensive attempt to document within-DTU diversity in *T. cruzi* to date. Nonetheless, some sample sizes remain limiting in population genetic terms, although efforts were made to correct for any confounding effects. Similarly, caution is required given the deviation of *T. cruzi* from the assumptions of most standard population genetic models due to clonality. Certainly, high levels of genetic diversity in the principal silvatic TcI populations examined in this study are consistent with the putative ancient (3–16 MYA) origin of this DTU [1]. Similarly, rare allele frequency plots are consistent with a large, stable *N_e_* [25]. Furthermore, we have shown that similar diversity indices could be derived from a study area of 10 km^2^ (*BOL*_North_) as from one of 4,500,000 km^2^ (*BRAZ*_North-East_), which suggests that this study has barely scraped the surface of the total circulating diversity present. In the silvatic environment, no apparent component of this diversity is partitioned by host. Thus, a constrained, extant co-evolutionary relationship is not compatible with the current dataset; contrary to a recent study using mini-exon sequence data from a limited number of *Didelphis* TcI strains [13]. Previously, we have suggested the ecological niche, rather than reservoir host, plays the dominant role in driving *T. cruzi* diversification [7]. This reflects a current model for wider trypanosome evolution, where “ecological host-fitting” is thought to define parasite clades [27]. Low levels of subdivision (*F*_ST_) between three populations sharing a similar ecotope across Amazonia are consistent with this supposition. While we demonstrate that TcI is eclectic in terms of host in arboreal lowland silvatic cycles, significant documentary evidence exists to suggest that *D. marsupialis* is the major carrier throughout much of lowland tropical South and Central America [7]. The majority of isolates examined here originate from this host. Tolerance by this species of high circulating parasitemia [28], as well as a possible propensity for non-vectorial transmission via infected territorial anal scent gland secretions [29], may predispose *D. marsupialis* to particularly intense *T. cruzi* transmission. Nonetheless, numerous vectors and secondary hosts are also implicated in TcI transmission and carriage [7],[30], and parasite dispersal between geographic foci is unlikely to be linked to *D. marsupialis* alone. Continental scale spatial structure in silvatic TcI (Figure 3) fits with the general ecology of undisturbed wild transmission. Most triatomine vectors, for example, are ill-adapted to long-range flight, and are thus incapable of rapid parasite dispersal between distant foci, providing ample time for spatial differentiation to occur among parasite populations.

Sample size corrected genetic diversity estimates suggest a considerable reduction in genetic differentiation in *AM*_North/Cen_ with respect to core silvatic populations. Furthermore, IBD breaks down among these isolates and a loss of rare alleles in this population could be interpreted as evidence of a recent population bottleneck [25]. Until recently, genetic studies of TcI diversity have failed to detect the signature of a rapid biogeographic expansion of this DTU into the USA [31]. Our findings are bolstered by low genetic diversity identified among new mini-exon sequence data derived from North and Central American TcI isolates [13], but greater sampling from this region would confirm our observations. The expansion of TcI into North and Central America is likely to have occurred since the formation of the Isthmus of Panama 2–4 MYA, providing a useful phylogeographic calibration point for future studies, and may correspond to the northerly migration of didelphid marsupials [32].

In this study, TcI strains from infected humans were sampled widely in Venezuela (Table S2). Although their sample size is currently limited (n = 15 for the domestic clade – includes one vector isolate (Table S2)), their robust genetic clustering, by comparison to the extensively sampled and genetically diverse parasite population from the silvatic environment, serves to make them representative and important. There are suggestions that Chagas disease is locally resurgent [33], and genetic discontinuity between the domestic population and most silvatic isolates raises significant questions regarding human disease transmission. Molecular data from the low-lying west of the country demonstrates that most silvatic and domestic populations of the principal vector, *Rhodnius prolixus*, are indistinguishable [34] and it follows that the parasite should also be invasive. However, in our study, the predominant *T. cruzi* strains infecting humans in the same and nearby areas bear little resemblance to those in the silvatic environment. Intriguingly, however, silvatic TcI genotypes prevail among almost all adult intradomiciliary triatomines sampled. All three triatomine species, *Triatoma maculata*, *Panstrongylus geniculatus*, and *R. prolixus* are also described from the silvatic environment in Venezuela [3] and could, therefore, be invasive, and the parasite strains infecting them not of human origin.

The occurrence of a domestic TcI clade in Venezuela, in spite of the presence of silvatic strains inside houses, presents an interesting problem. Among African trypanosomes (*T. brucei sspp.*), human infective forms display only a limited array of genotypes (*T. b. rhodesiense & gambiense* [20],[35]). Detailed studies of *T. b. brucei* population genetics in the silvatic environment are, however, lacking. Some evidence suggests that vectors and domestic mammalian reservoirs in *T. b. brucei* populations sympatric with human *T. b. rhodesiense* outbreaks support a greater diversity of strains [20]. However, no specific genes associated with human infectivity are known in *T. cruzi*, unlike in *T. b. rhodesiense* [36], that might drive the domestic expansion of an epidemic clone. Furthermore, silvatic-type TcI strains were capable of sustaining long-term, symptomatic infection in a subset of patients studied (Table S2). One possible confounder in our sampling, as in a recent population study of strains from West African *T. b. gambiense* symptomatic human infections [35], is a lack of samples from asymptomatic patients, which are required to refute an association between parasite genotype and virulence or pathogenicity.

In the absence of a clear adaptive explanation for the lack of diversification among Venezuelan domestic isolates on the basis of current data, an ecological one may be more parsimonious. Low transmission of the parasite to the human host by invasive adult triatomines may reflect the inefficient stercorarian route by which *T. cruzi* is normally spread [2]. Instead, repeated blood meals taken by domestic triatomine colonies may be necessary to ensure infective contact with the human host. In this case, other humans or domestic reservoirs will be the primary sources of human infection, human and domestic vector migration the main driver of parasite dispersal, and a widespread, uniform domestic parasite genotype the result. This is an observation supported by a lack of IBD among domestic strains. The distribution of this genotype may be wider than described here, and there is now preliminary mini-exon sequence evidence that a domestic TcI genotype may also occur in Colombia [14].

The origin of the divergent Venezuelan human TcI population remains enigmatic. Isolates bear closest resemblance, by all measures employed in this study, to the North and Central American clade. In all likelihood, TcI populations migrated to the North prehistorically in conjunction with invasive mammalian reservoir hosts during the Great American Interchange [32]. Low genetic diversity is also identified in domestic *R. prolixus* populations from Central America [37], although presumably their northerly migration occurred many thousands of years later alongside human populations. It is highly improbable that domestic TcI strains carried northwards with *R. prolixus* subsequently dispersed so widely into the silvatic environment. The source of the domestic outbreak identified here probably remains sequestered among silvatic transmission cycles somewhere in the northerly distribution of TcI in South America.

A greater sampling effort is required around Cochabamba (*ANDES*_Bol_) from both human and wild reservoirs before satisfactory conclusions can be drawn regarding local parasite transmission. Intriguingly, temporal heterogeneity seems to be negligible, and ∼20 years separate the isolation of human and rodent strains (Table S2). Epidemiologically, congruence between populations from these two hosts is not unexpected. Local domestic and silvatic *T. infestans* populations match genetically and morphologically [38], and rodent isolates were collected within two kilometres of a major suburb of Cochabamba, where active urban transmission still occurs [39]. It is not clear, however, whether the parasite is invasive to the domestic setting, or whether domestic strains have re-invaded the silvatic cycle.

A major observation of this study, and in others examining genetic diversity in *T. cruzi* [1],[4],[15], is the deficiency of heterozygosity with respect to Hardy-Weinberg expectations observed in most populations. Similar observations are frequently made in the *Leishmania spp.* populations [40]–[42]. These levels of homozygosity are atypical with respect to other clonally reproducing diploids [35],[43],[44], where diversity is known to accumulate between alleles within the individual in the absence of recombination, leading to extreme levels of heterozygosity at homologous loci (the ‘Meselson effect’ [45]). Heterozygous deficiency in silvatic populations in our dataset cannot be uniquely attributed to hidden subdivision (Walhund effect). We still find positive *F*_IS_ values in non-subdivided sub-samples of isolates within populations. Here, some increase in heterozygosity was observed (Figure 2), but not to the extent predicted by the Meselson effect. Multilocus linkage disequilibrium suggests that recombination is at most infrequent in the current dataset, although the Index of Association [46] is a relatively insensitive measure [44]. Thus, widespread loss of heterozygosity due to homologous recombination or gene conversion, not inbreeding, is the most likely genetic phenomenon that would result in the observed diversity in our data. Importantly, we can show that these events are apparently genomically diffuse, in silvatic populations at least. Most SSFs show similar levels of heterozygosity within populations, rather than some showing strong evidence of the Meselson effect (strongly negative *F*_IS_) and others showing complete homozygosity, as would be expected of larger scale effects like ploidy cycles [47] or those following genome fusion events in yeast [48].

Populations *ANDES*_Bol/Chil_ and *VEN*_Dom_ share many features in population genetic terms: reduced diversity; non-equilibrium rare allele frequencies; and high inter-SSF variance in *F*_IS_ values where strongly negative values on some SSFs reflect marginally raised overall heterozygosity at the population level. It remains to be seen whether these are unique characteristics of human TcI clades, whether they reflect possible past recombination events or some form of balancing selection, and we could not attribute significance to a decrease in *F*_IS_ from background levels. DTUs TcIId and TcIIe both show fixed heterozygosity at most loci because they are almost certainly hybrids [1],[5], not due to the Meselson effect, and far in excess of heterozygosity levels observed in our dataset. Confirmation of the characteristics we have observed will come with more intensive sampling from domestic foci in both regions, as well as others across South America. Our data now show, with increasing support from other studies [13],[14],[49],[50], that most *T. cruzi* lineages actually represent highly heterogeneous populations across their distribution, heterogeneity that may be highly informative in epidemiological terms. Control strategies would now greatly benefit from high density parasitological surveys in and around individual endemic disease foci, especially if a pathogenic human TcI genotype does exist, signalling a return in study design, if not methodology, to the early investigations of the 1970s [21]. Such studies should include parasite samples from silvatic mammals and vectors, as well as domestic sources, including both symptomatic and asymptomatic (or indeterminate) human cases. To this extent, using microsatellite markers developed here, *T. cruzi* population genetics can be observed at the finest scale and provide real insights into the true nature of Chagas disease transmission.

## Methods

We assembled a panel of 135 *T. cruzi* samples belonging to TcI from throughout the silvatic distribution of this lineage (Table S2). DTU-level genotyping was achieved through analysis of the non-transcribed spacer region of the mini-exon gene, as described previously [51]. Microsatellite motifs were extracted from the draft sequence of the *T. cruzi* genome available at http://www.genedb.org. Four Mb of sequence, including at least 13 syntenous sequence fragments, were scanned for di- and tri-nucleotide repeats using a pattern matching script written in *sed*. An extension of the algorithm was included to extract the up and downstream flanking regions of the microsatellite sequence (∼200 bp). Primer design was achieved in PRIMER3 [52].

Among 200 microsatellite loci identified, 45 were polymorphic. A further three were included from two previous studies [6],[15]. Primers and binding sites are listed in Table S3. The following reaction cycle was implemented across all loci: a denaturation step of 4 minutes at 95°C, then 30 amplification cycles (95°C for 20 seconds, 57°C for 20 seconds, 72°C for 20 seconds) and a final 20 minute elongation step at 72°C. With a final volume of 10 ul, 1× ThermoPol Reaction Buffer (New England Biolabs (NEB), UK), 4 mM MgCl_2_, 34 uM dNTPs; 0.75 pmols of each primer, 1 unit of *Taq* polymerase (NEB, UK) and 1 ng of genomic DNA were added. Five fluorescent dyes were used to label forward primers – 6-FAM & TET (Proligo, Germany), NED, PET & VIC (Applied Biosystems, UK). Allele sizes were determined using an automated capillary sequencer (AB3730, Applied Biosystems, UK), manually checked for errors and typed “blind” to control for user bias.

### Microsatellite diversity analysis

Allelic richness estimates were calculated in FSTAT 2.9.3.2 [53] and corrected for sample size using Hurlbert's rarefaction method [54] in MolKin v3.0 [55]. Pair-wise estimates of population subdivision (*F*_ST_, Table S1) and heterozygosity indices (Table 1) were estimated in ARLEQUIN 3.0 [56]. P-values for multiple tests were corrected using a sequential Bonferroni correction [57]. *F*_IS_ provides an alternative measure of heterozygosity by assessing the level of identity of alleles within individuals compared to that between individuals where +1 represents all individuals homozygous for different alleles, and −1 all individuals heterozygous for the same alleles. Mean *F*_IS_ estimates over loci in selected groups of sub-populations were calculated in FSTAT 2.9.3.2 using Weir and Cockerman's (1984) unbiased estimators [58]. Confidence intervals for *F*_IS_ estimates were calculated by bootstrapping over loci and tests for significant differences between values also in FSTAT 2.9.3.2 using 10,000 random permutations. Mean *F*_IS_ values per sequence fragment per population were calculated across standard (not Weir and Cockerman's) *F*_IS_ values in FSTAT 2.9.3.2. To assess the level of multilocus linkage disequilibrium, the Index of Association (I_A_, multilocus) was calculated in MULTILOCUS 1.3b [46],[59] (Table 1). Genetic distances between isolates were evaluated in MICROSAT under an infinite alleles model of microsatellite evolution using *D*_AS_ (1-proportion of shared alleles at all loci / *n*) [60] (Figure 1). To accommodate multi-allelic loci, a script was written in Microsoft Visual Basic to make multiple random diploid re-samplings of each multilocus profile (software available on request). Individual-level genetic distances were calculated as the mean across multiple re-sampled datasets. A single randomly sampled dataset was used for population-level analysis. A Mantel's test for matrix correspondence was executed in GENALEX 6 to compare pair-wise geographical (km) and genetic distance (*D*_AS_) [61] (Figure 3). Samples were assigned to populations on an *a priori* basis according to geography and transmission cycle. *D*_AS_ - defined sample clustering was also used to inform population identity, and obvious outliers assigned to the correct genetic group (Figure 1). Rare allele frequency plots were calculated as in Luikart *et al.*, 1998 [25], to detect perturbation following putative population events (e.g. population bottlenecks).

## Supporting Information

Figure S1 Allele frequency classes among seven TcI populations.
(6.79 MB TIF)

Table S1 FST estimates of interpopulation differentiation for seven TcI subpopulations based on microsatellite data.
(0.04 MB DOC)

Table S2 Panel of *T. cruzi* TcI genotype isolates assembled for microsatellite analysis.
(0.31 MB DOC)

Table S3 Microsatellite loci and primers employed in this study.
(0.12 MB DOC)
