# Supplementary figures and images for: Genome-Scale Multilocus Microsatellite Typing of Trypanosoma cruzi Discrete Typing Unit I Reveals Phylogeographic Structure and Specific Genotypes Linked to Human Infection
Source: PLoS Pathog. 2009 May 1;5(5):e1000410. doi: 10.1371/journal.ppat.1000410 (PMC2669174; doi:10.1371/journal.ppat.1000410)

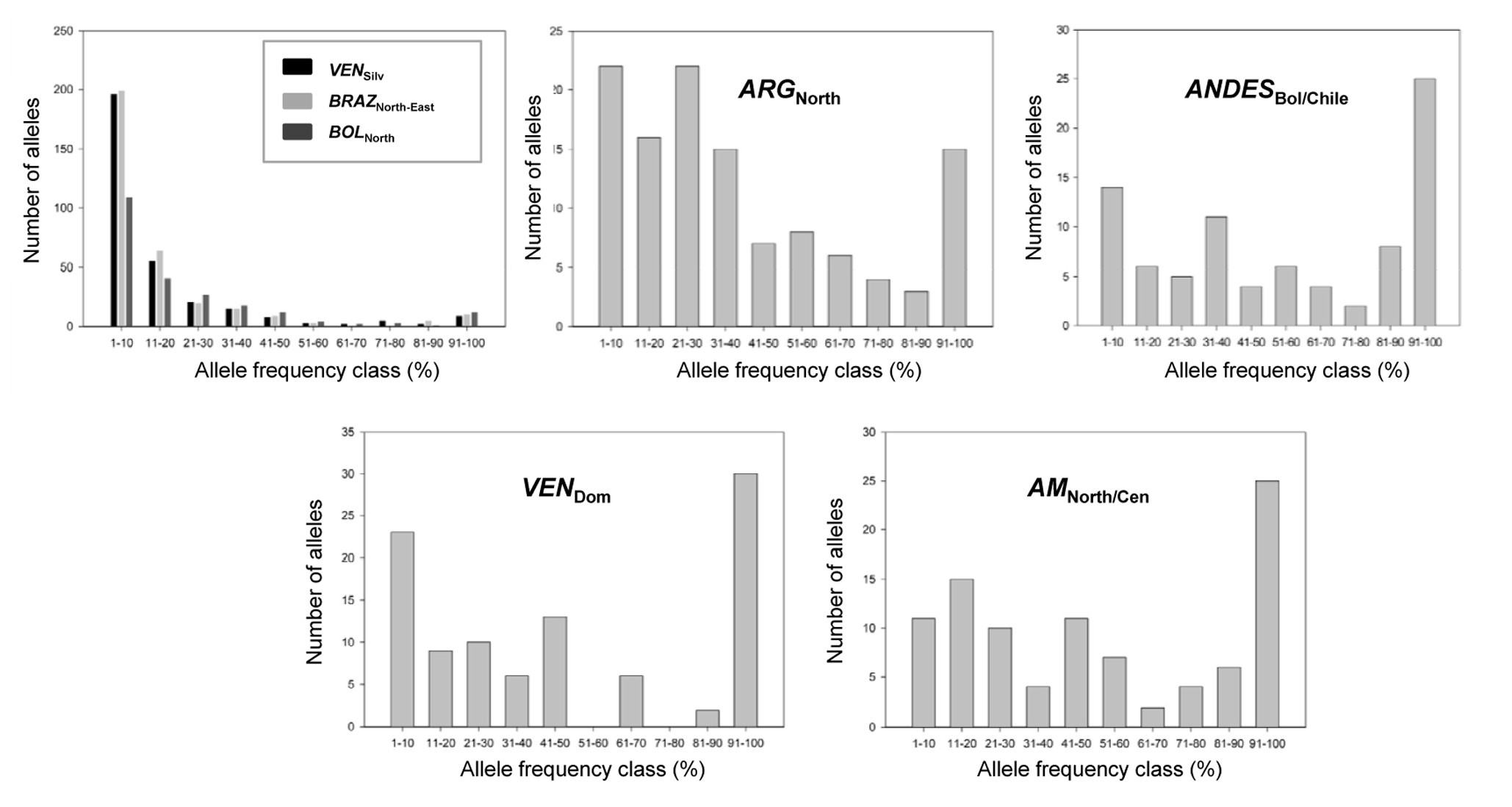

Supplement: Figure S1 — Allele frequency classes among seven TcI populations. (6.79 MB TIF) [file ppat.1000410.s001.tif]
