# Supplementary material for: Genome-Scale Multilocus Microsatellite Typing of Trypanosoma cruzi Discrete Typing Unit I Reveals Phylogeographic Structure and Specific Genotypes Linked to Human Infection
Source: PLoS Pathog. 2009 May 1;5(5):e1000410. doi: 10.1371/journal.ppat.1000410 (PMC2669174; doi:10.1371/journal.ppat.1000410)
Supplement: Table S1 — FST estimates of interpopulation differentiation for seven TcI subpopulations based on microsatellite data. (0.04 MB DOC) [file ppat.1000410.s002.doc]

Table S1 *F*_ST_ estimates of inter-population differentiation for seven TcI subpopulations based on microsatellite data.

|  | *ARG*_North_ | *ANDES*_Bol/Chile_ | *BOL*_North_ | *BRAZ*_North-East_ | *AM*_North/Cen_ | *VEN*_dom_ | *VEN*_silv_ |
| --- | --- | --- | --- | --- | --- | --- | --- |
| *ARG*_North_ | * | *<0.00001* | *<0.00001* | *<0.00001* | *<0.00001* | *<0.00001* | *<0.00001* |
| *ANDES*_Bol/Chile_ | 0.343 | * | *<0.00001* | *<0.00001* | *<0.00001* | *<0.00001* | *<0.00001* |
| *BOL*_North_ | 0.207 | 0.304 | * | *<0.00001* | *<0.00001* | *<0.00001* | *<0.00001* |
| *BRAZ*_North-East_ | 0.205 | 0.319 | 0.144 | * | *<0.00001* | *<0.00001* | *<0.00001* |
| *AM*_North/Cen_ | 0.489 | 0.650 | 0.340 | 0.278 | * | *<0.00001* | *<0.00001* |
| *VEN*_dom_ | 0.570 | 0.711 | 0.411 | 0.356 | 0.251 | * | *<0.00001* |
| *VEN*_silv_ | 0.226 | 0.330 | 0.148 | 0.108 | 0.239 | 0.295 | * |

Italics indicate p-values generated from 1000 random permutations leading to a value larger than or equal to that observed. All values remain significant after sequential Bonferroni correction**.**
