# Supplementary material for: Genome-Scale Multilocus Microsatellite Typing of Trypanosoma cruzi Discrete Typing Unit I Reveals Phylogeographic Structure and Specific Genotypes Linked to Human Infection
Source: PLoS Pathog. 2009 May 1;5(5):e1000410. doi: 10.1371/journal.ppat.1000410 (PMC2669174; doi:10.1371/journal.ppat.1000410)
Supplement: Table S2 — Panel of T. cruzi TcI genotype isolates assembled for microsatellite analysis. (0.31 MB DOC) [file ppat.1000410.s003.doc]

Table S2 Panel of *T. cruzi* TcI genotype isolates assembled for microsatellite analysis.

| **Host/Vector** | **Original Code^a^** | **Date** | **Locality** | **Latitude^b^** | **Longitude^b^** | **Population^c^** | **Source^d^** |
| --- | --- | --- | --- | --- | --- | --- | --- |
|
| *Didelphis marsupialis* | **92090802P** | 1992 | Georgia. USA | 32.43 | -83.31 | *AM*_North/Cen_ | IRD |
| *Didelphis marsupialis* | **93070103P** | 1993 | Georgia. USA | 32.43 | -83.31 | *AM*_North/Cen_ | IRD |
| *Didelphis marsupialis* | **92101601P** | 1992 | Georgia, USA | 32.43 | -83.31 | *AM*_North/Cen_ | IRD |
| *Triatoma dimidiata* | DAVIS 9.90 | 1983 | Tegucigalpa, Honduras | 14.08 | -87.2 | *AM*_North/Cen_ | IRD |
| *Triatoma sanguisuga* | FLORIDAC1D12 | Unknown | Florida, USA | 30.5 | -81.66 | *AM*_North/Cen_ | IRD |
| *Didelphis marsupialis* | **USAOPOSSUM** | Unknown | Lousiana, USA | 30.5 | -91 | *AM*_North/Cen_ | IRD |
| *Dasypus novemcinctus* | **USAARMA** | Unknown | Lousiana, USA | 30.5 | -91 | *AM*_North/Cen_ | IRD |
| *Mepraia spinolai* | **CHILEC22** | c.1984 | Flor de Valle, Chile | -30.31 | -71.22 | *ANDES*_Bol/Chile_ | LSHTM |
| *Mepraia spinolai* | **CHILEWALL** | c.1984 | Flor de Valle, Chile | -30.31 | -71.22 | *ANDES*_Bol/Chile_ | LSHTM |
| *Akodon boliviensis* | COTMA22^4^ | 11.10.04 | Cotopachi, Bolivia | -17.43 | -66.27 | *ANDES*_Bol/Chile_ | ML |
| *Akodon boliviensis* | COTMA38^4^ | 13.10.04 | Cotopachi, Bolivia | -17.43 | -66.27 | *ANDES*_Bol/Chile_ | ML |
| *Phyllotis ocilae* | COTMA47^4^ | 13.10.04 | Cotopachi, Bolivia | -17.43 | -66.27 | *ANDES*_Bol/Chile_ | ML |
| *Phyllotis ocilae* | COTMA55^4^ | 14.10.04 | Cotopachi, Bolivia | -17.43 | -66.27 | *ANDES*_Bol/Chile_ | ML |
| *Phyllotis ocilae* | COTMA9^4^ | 10.10.04 | Cotopachi, Bolivia | -17.43 | -66.27 | *ANDES*_Bol/Chile_ | ML |
| *Homo sapiens* | P234^4^ | 1985 | Cochabamba, Bolivia | -17.38 | -66.16 | *ANDES*_Bol/Chile_ | IRD |
| *Homo sapiens* | P238^4^ | 1985 | Cochabamba, Bolivia | -17.38 | -66.16 | *ANDES*_Bol/Chile_ | IRD |
| *Homo sapiens* | P268^4^ | 1987 | Cochabamba, Bolivia | -17.38 | -66.16 | *ANDES*_Bol/Chile_ | IRD |
| *Homo sapiens* | PII(BOL)^4^ | c.1980 | Cochabamba, Bolivia | -17.38 | -66.16 | *ANDES*_Bol/Chile_ | IRD |
| *Didelphis albiventris* | **PALDA1** | 23.3.01 | Chaco, Argentina | -27.13 | -61.46 | *ARG*_North_ | PD |
| *Didelphis albiventris* | **PALDA20** | 23.3.01 | Chaco, Argentina | -27.13 | -61.46 | *ARG*_North_ | PD |
| *Didelphis albiventris* | PALDA21 | 23.3.01 | Chaco, Argentina | -27.133 | -61.46 | *ARG*_North_ | PD |
| *Didelphis albiventris* | **PALDA22** | 23.3.01 | Chaco, Argentina | -27.133 | -61.46 | *ARG*_North_ | PD |
| *Didelphis albiventris* | **PALDA3** | 23.3.01 | Chaco, Argentina | -27.133 | -61.46 | *ARG*_North_ | PD |
| *Didelphis albiventris* | **PALDA4** | 23.3.01 | Chaco, Argentina | -27.133 | -61.46 | *ARG*_North_ | PD |
| *Didelphis albiventris* | **PALDA5** | 23.3.01 | Chaco, Argentina | -27.133 | -61.46 | *ARG*_North_ | PD |
| *Triatoma infestans* | PALDAV2^3* | 23.3.01 | Chaco, Argentina | -27.133 | -61.46 | *ARG*_North_ | PD |
| *Didelphis albiventris* | **TEDA** | 23.3.01 | Chaco, Argentina | -26.93 | -61.58 | *ARG*_North_ | PD |
| *Triatoma infestans* | TEV55* | 23.3.01 | Chaco, Argentina | -26.93 | -61.58 | *ARG*_North_ | PD |
| *Didelphis marsupialis* | SJM18^1^ | 5.9.04 | Beni, Bolivia | -14.81 | -64.6 | *BOL*_North_ | ML |
| *Didelphis marsupialis* | **SJM22^1^** | 6.9.04 | Beni, Bolivia | -14.81 | -64.6 | *BOL*_North_ | ML |
| *Didelphis marsupialis* | SJM23^1^ | 6.9.04 | Beni, Bolivia | -14.81 | -64.6 | *BOL*_North_ | ML |
| *Didelphis marsupialis* | SJM26^1^ | 6.9.04 | Beni, Bolivia | -14.81 | -64.6 | *BOL*_North_ | ML |
| *Didelphis marsupialis* | SJM3^2^ | 2.9.04 | Beni, Bolivia | -14.81 | -64.6 | *BOL*_North_ | ML |
| *Philander opossum* | SJM32^2^ | 7.9.04 | Beni, Bolivia | -14.81 | -64.6 | *BOL*_North_ | ML |
| *Didelphis marsupialis* | SJM33^1^ | 7.9.04 | Beni, Bolivia | -14.81 | -64.6 | *BOL*_North_ | ML |
| *Didelphis marsupialis* | **SJM34^1^** | 7.9.04 | Beni, Bolivia | -14.81 | -64.6 | *BOL*_North_ | ML |
| *Didelphis marsupialis* | SJM35^2^ | 7.9.04 | Beni, Bolivia | -14.81 | -64.6 | *BOL*_North_ | ML |
| *Didelphis marsupialis* | **SJM37^2^** | 9.9.04 | Beni, Bolivia | -14.81 | -64.6 | *BOL*_North_ | ML |
| *Didelphis marsupialis* | **SJM39^1^** | 9.9.04 | Beni, Bolivia | -14.81 | -64.6 | *BOL*_North_ | ML |
| *Didelphis marsupialis* | SJM40^2^ | 9.9.04 | Beni, Bolivia | -14.81 | -64.6 | *BOL*_North_ | ML |
| *Philander opossum* | **SJM41^2^** | 9.9.04 | Beni, Bolivia | -14.81 | -64.6 | *BOL*_North_ | ML |
| *Philander opossum* | SJMC12^2^ | 13.9.04 | Beni, Bolivia | -14.81 | -64.6 | *BOL*_North_ | ML |
| *Didelphis marsupialis* | **SJMC3^1^** | 6.9.04 | Beni, Bolivia | -14.81 | -64.6 | *BOL*_North_ | ML |
| *Scuireus spadiceus* | SJMC7^2^ | 11.9.04 | Beni, Bolivia | -14.81 | -64.6 | *BOL*_North_ | ML |
| *Potus flavus* | **458** | 1987 | Bajo Calima, Colombia | 3.98 | -76.96 | *BRAZ*_North-East_ | IRD |
| *Philander opossum* | B1947 | 28.01.92 | Barcarena, Brazil | -1.5 | -48.18 | *BRAZ*_North-East_ | EC |
| *Philander opossum* | **B2026** | 09.05.89 | Camatá, Brazil | -2.61 | -49.53 | *BRAZ*_North-East_ | EC |
| *Philander opossum* | B2077 | 13.12.90 | Belem, Brazil | -1.36 | -48.36 | *BRAZ*_North-East_ | EC |
| *Didelphis marsupialis* | B2310 | 9.12.94 | Afua, Brazil | -0.15 | -50.38 | *BRAZ*_North-East_ | EC |
| *Didelphis marsupialis* | B2311 | 9.12.94 | Afua, Brazil | -0.15 | -50.38 | *BRAZ*_North-East_ | EC |

**Table S2** Continued from overleaf.

| *Didelphis marsupialis* | **XE5740** | 18.6.02 | Para, Brazil | -1.38 | -48.86 | *BRAZ*_North-East_ | EC |
| --- | --- | --- | --- | --- | --- | --- | --- |
| *Didelphis marsupialis* | CJO20 | c.1994 | Carajas, Brazil | -5.98 | -51.33 | *BRAZ*_North-East_ | HC |
| *Didelphis marsupialis* | CJO33 | c.1994 | Carajas, Brazil | -5.98 | -51.33 | *BRAZ*_North-East_ | HC |
| *Didelphis marsupialis* | IM4810 | 23.4.02 | Manaus, Brazil | -3.07 | -60.16 | *BRAZ*_North-East_ | EC |
| *Philander opossum* | **XE1313** | 23.11.83 | Carajas, Brazil | -5.98 | -51.33 | *BRAZ*_North-East_ | EC |
| *Didelphis marsupialis* | XE1342 | 29.11.83 | Carajas, Brazil | -5.98 | -51.33 | *BRAZ*_North-East_ | EC |
| *Didelphis marsupialis* | XE1381 | 16.12.83 | Carajas, Brazil | -5.98 | -51.33 | *BRAZ*_North-East_ | EC |
| *Didelphis marsupialis* | XE1383 | 16.12.83 | Carajas, Brazil | -5.98 | -51.33 | *BRAZ*_North-East_ | EC |
| *Didelphis marsupialis* | B2084 | 13.12.90 | Belem, Brazil | -1.36 | -48.36 | *BRAZ*_North-East_ | EC |
| *Didelphis marsupialis* | B2085 | 03.01.91 | Belem, Brazil | -1.36 | -48.36 | *BRAZ*_North-East_ | EC |
| *Philander opossum* | XE2913 | 05.05.88 | Belem, Brazil | -1.36 | -48.36 | *BRAZ*_North-East_ | EC |
| *Didelphis marsupialis* | XE2929 | 10.08.88 | Pará, Brazil | -5.83 | -48.03 | *BRAZ*_North-East_ | EC |
| *Philander opossum* | B3159 | 16.6.89 | Pará, Brazil | -2.32 | -49.52 | *BRAZ*_North-East_ | EC |
| *Didelphis marsupialis* | XE3309 | 13.12.90 | Belem, Brazil | -1.36 | -48.366 | *BRAZ*_North-East_ | EC |
| *Marmosa cinerea* | B3726 | 10.6.98 | Amapa, Brazil | 0.03 | -51.05 | *BRAZ*_North-East_ | EC |
| *Didelphis marsupialis* | XE3776 | 23.10.98 | Para, Brazil | -1.51 | -49.21 | *BRAZ*_North-East_ | EC |
| *Didelphis marsupialis* | XE3981 | 09.01.95 | Para, Brazil | -0.15 | -50.383 | *BRAZ*_North-East_ | EC |
| *Didelphis marsupialis* | XE4389 | 16.05.96 | Carajás, Brazil | -5.98 | -51.33 | *BRAZ*_North-East_ | EC |
| *Didelphis marsupialis* | XE4682 | Unknown | Carajás, Brazil | -1.51 | -49.21 | *BRAZ*_North-East_ | EC |
| *Philander opossum* | XE4993 | 20.8.01 | Para, Brazil | -3.66 | -49.63 | *BRAZ*_North-East_ | EC |
| *Didelphis marsupialis* | XE5011 | 13.01.99 | Para, Brazil | -1.05 | -46.76 | *BRAZ*_North-East_ | EC |
| *Didelphis marsupialis* | XE5012 | 13.01.99 | Para, Brazil | -1.05 | -46.76 | *BRAZ*_North-East_ | EC |
| *Didelphis marsupialis* | XE5017 | 13.01.99 | Para, Brazil | -1.05 | -46.76 | *BRAZ*_North-East_ | EC |
| *Didelphis marsupialis* | XE5164 | 14.09.99 | Para, Brazil | -1.71 | -48.88 | *BRAZ*_North-East_ | EC |
| *Didelphis marsupialis* | **XE5165** | 14.09.99 | Para, Brazil | -1.71 | -48.88 | *BRAZ*_North-East_ | EC |
| *Didelphis marsupialis* | **XE5167** | 14.09.99 | Para, Brazil | -1.71 | -48.88 | *BRAZ*_North-East_ | EC |
| *Didelphis marsupialis* | B5770 | 4.13.99 | Para, Brazil | -1.05 | -46.76 | *BRAZ*_North-East_ | EC |
| *Didelphis marsupialis* | B5781 | 16.9.99 | Para, Brazil | -1.71 | -48.88 | *BRAZ*_North-East_ | EC |
| *Didelphis marsupialis* | B5787 | 16.9.99 | Para, Brazil | -1.71 | -48.88 | *BRAZ*_North-East_ | EC |
| *Didelphis marsupialis* | XE5809 | 23.5.02 | Tocantins, Brazil | -9.18 | -48.183 | *BRAZ*_North-East_ | EC |
| *Didelphis marsupialis* | XE5847 | 12.11.01 | Para, Brazil | -1.38 | -48.86 | *BRAZ*_North-East_ | EC |
| *Philander opossum* | XE5495 | 21.02.01 | Para, Brazil | -1.86 | -52.2 | *BRAZ*_North-East_ | EC |
| *Didelphis marsupialis* | XE6004 | 17.10.03 | Roraima, Brazil | 2.76 | -60.53 | *BRAZ*_North-East_ | EC |
| *Homo sapiens* | 7570^e^ (67/M) | 1998 | Tachira, Venezuela | 7.76 | -72.25 | *VEN*_dom_ | HC |
| *Homo sapiens* | 8104^e, f^  (51/F) | 1999 | Miranda, Venezuela | 10.23 | -66.66 | *VEN*_dom_ | HC |
| *Homo sapiens* | 9354^e^ (70/M) | 1999 | Sucre, Venezuela | 10.46 | -63.61 | *VEN*_dom_ | HC |
| *Homo sapiens* | 10775^e^ (65/F) | 2002 | Barinas, Venezuela | 8.37 | -70.51 | *VEN*_dom_ | HC |
| *Homo sapiens* | 10801^e^ (65/M) | 2002 | Guarico, Venezuela | 8.91 | -65.38 | *VEN*_dom_ | HC |
| *Homo sapiens* | 11006^e^ (61/F) | 2002 | Cojedes, Venezuela | 9.66 | -68.2 | *VEN*_dom_ | HC |
| *Homo sapiens* | 11042^e^ (55/F) | 2002 | Portuguesa, Venezuela | 8.66 | -69.5 | *VEN*_dom_ | HC |
| *Homo sapiens* | 11124^e^ (57/F) | 2002 | Dtto Federal, Venezuela | 10.61 | -67.04 | *VEN*_dom_ | HC |
| *Homo sapiens* | 11398 | 2003 | Merida, Venezuela | 8.59 | -71.23 | *VEN*_dom_ | HC |
| *Homo sapiens* | 11541^e^ (58/M) | 2003 | Merida, Venezuela | 8.59 | -71.23 | *VEN*_dom_ | HC |
| *Homo sapiens* | 11713^e^ (53/M) | 2003 | Lara, Venezuela | 10.04 | -69.32 | *VEN*_dom_ | HC |
| *Homo sapiens* | 11804^e, f^  55(49/M)5 | 2003 | Portuguesa, Venezuela | 9.01 | -69.29 | *VEN*_dom_ | HC |
| *Homo sapiens* | 11838 | 2003 | Guarico, Venezuela | 8.71 | -66.62 | *VEN*_dom_ | HC |
| *Homo sapiens* | 11881^e^ (51/M) | 2003 | Anzoategui, Venezuela | 9.36 | -65.12 | *VEN*_dom_ | HC |
| *Rhodnius prolixus* | CASABONIF* | 2001 | Portuguesa, Venezuela | 9.01 | -69.29 | *VEN*_dom_ | HC |
| *Homo sapiens* | 9010^e^ (56/M) | 1999 | Trujillo,Venezuela | 9.55 | -70.51 | *VEN*_silv_ | HC |
| *Didelphis marsupialis* | 361TA | 1986 | Guadual, Colombia | 1.71 | -77.91 | *VEN*_silv_ | IRD |

**Table S2 Continued from overleaf.**

| *Didelphis marsupialis* | AM3 | 2000 | Sucre, Venezuela | 10.11 | -64.55 | *VEN*_silv_ | HC |
| --- | --- | --- | --- | --- | --- | --- | --- |
| *Didelphis marsupialis* | AM6 | 2000 | Sucre, Venezuela | 10.11 | -64.55 | *VEN*_silv_ | HC |
| *Rhodnius prolixus* | AMRPAE4 | 2005 | Anzoategui, Venezuela | 9.01 | -64.34 | *VEN*_silv_ | HC |
| *Triatoma maculata* | BARM104** | 2004 | Barinas, Venezuela | 8.37 | -70.51 | *VEN*_silv_ | HC |
| *Rhodnius prolixus* | CALC104 | 2004 | Carabobo, Venezuela | 10.19 | -68 | *VEN*_silv_ | HC |
| *Didelphis marsupialis* | CD45 | 1999 | Distrito Federal, Venezuela | 10.35 | -67.03 | *VEN*_silv_ | LSHTM |
| *Rattus rattus* | C022 | 1994 | Distrito Federal, Venezuela | 10.54 | -67.8 | *VEN*_silv_ | HC |
| *Didelphis marsupialis* | CO57 | 1994 | Distrito Federal, Venezuela | 10.54 | -67.8 | *VEN*_silv_ | HC |
| *Didelphis marsupialis* | CO75 | 1995 | Distrito Federal, Venezuela | 10.54 | -67.8 | *VEN*_silv_ | HC |
| *Rattus rattus* | CO84 | 1995 | Miranda, Venezuela | 10.31 | -66.39 | *VEN*_silv_ | HC |
| *Didelphis marsupialis* | DM1 | 1997 | Trujillo, Venezuela | 9.55 | -70.51 | *VEN*_silv_ | HC |
| *Didelphis marsupialis* | DM4 | 1997 | Trujillo, Venezuela | 9.55 | -70.51 | *VEN*_silv_ | HC |
| *Didelphis marsupialis* | DMSU8 | 2004 | Sucre, Venezuela | 10.46 | -63.61 | *VEN*_silv_ | HC |
| *Didelphis marsupialis* | DMSUC | 2001 | Sucre, Venezuela | 10.46 | -63.61 | *VEN*_silv_ | HC |
| *Homo sapiens* | **JR^e^ (55/M)** | 2001 | Anzoategui, Venezuela | 9.01 | -64.34 | *VEN*_silv_ | HC |
| *Rhodnius prolixus* | LL2LA^3^ | 15.5.04 | Barinas, Venezuela | 7.5 | -71.23 | *VEN*_silv_ | ML |
| *Didelphis marsupialis* | M12^3^ | 10.6.04 | Barinas, Venezuela | 7.5 | -71.23 | *VEN*_silv_ | ML |
| *Didelphis marsupialis* | **M13^3^** | 12.6.04 | Barinas, Venezuela | 7.5 | -71.23 | *VEN*_silv_ | ML |
| *Didelphis marsupialis* | M15^3^ | 13.6.04 | Barinas, Venezuela | 7.5 | -71.23 | *VEN*_silv_ | ML |
| *Didelphis marsupialis* | **M16^3^** | 13.6.04 | Barinas, Venezuela | 7.5 | -71.23 | *VEN*_silv_ | ML |
| *Didelphis marsupialis* | **M18^3^** | 13.6.04 | Barinas, Venezuela | 7.5 | -71.23 | *VEN*_silv_ | ML |
| *Didelphis marsupialis* | **M7^3^** | 14.5.04 | Barinas, Venezuela | 7.5 | -71.23 | *VEN*_silv_ | ML |
| *Didelphis marsupialis* | **PARAMA39** | 6.8.05 | Barinas, Venezuela | 8.43 | -70.55 | *VEN*_silv_ | ML |
| *Didelphis marsupialis* | PARAMA40 | 6.8.05 | Barinas, Venezuela | 8.43 | -70.55 | *VEN*_silv_ | ML |
| *Didelphis marsupialis* | PARAMA41 | 6.8.05 | Barinas, Venezuela | 8.43 | -70.55 | *VEN*_silv_ | ML |
| *Triatoma maculata* | PGN900** | 2000 | Miranda, Venezuela | 10.36 | -66.75 | *VEN*_silv_ | HC |
| *Rattus rattus* | RR5 | 2001 | Trujillo, Venezuela | 9.55 | -70.51 | *VEN*_silv_ | HC |
| *Rhodnius prolixus* | SANRAFLB | 16.5.04 | Barinas, Venezuela | 8.48 | -70.73 | *VEN*_silv_ | ML |
| *Rhodnius prolixus* | SANRAFP2A | 4.5.04 | Barinas, Venezuela | 8.48 | -70.73 | *VEN*_silv_ | ML |
| *Rhodnius prolixus* | SANRAFP2B | 4.5.04 | Barinas, Venezuela | 8.48 | -70.73 | *VEN*_silv_ | ML |
| *Rhodnius prolixus* | TCSCII | 2000 | Cojedes, Venezuela | 9.828 | -68.43 | *VEN*_silv_ | HC |
| *Rhodnius prolixus* | TERF-1 | 2001 | Portuguesa, Venezuela | 8.34 | -68.68 | *VEN*_silv_ | HC |
| *Panstrongylus geniculatus* | V1** | 1999 | Anzoategui, Venezuela | 10.04 | -64.32 | *VEN*_silv_ | HC |
| *Triatoma maculata* | V2** | 1999 | Anzoategui, Venezuela | 10.04 | -64.32 | *VEN*_silv_ | HC |
| *Rhodnius prolixus* | V4** | 2000 | Anzoategui, Venezuela | 10.04 | -64.32 | *VEN*_silv_ | HC |

^a^ Sample codes in bold represent strains biologically cloned in this study.

^b^ Decimal degrees

^c^ Population code

^d^ **IRD** – Institut de Recherche pour le Developpement, Montpellier, France, courtesy of C. Barnabe. **LSHTM** – held at LSHTM, London. **ML** – M. Llewellyn, LSHTM, London, **HC** – H. Carrasco, Universidad Central de Venezuela, Caracas. **PD** – P. Diosque, Universidad de Salta, Argentina, **EC –** Isolated by M. Miles, held at the Instituto Evandro Chagas, Belem, Para, Brazil

^e^ Known to be in the chronic, symptomatic phase of Chagas disease.

^f^ Not included in population level analyses (Table 1) due to DNA availability issues.

Number is parentheses: age/sex

*Originates from a domestically caught triatomine vector.

** Indicates a domestically caught triatomine vector from Venezuela infected with a silvatic-type strain (see text for details)

^1, 2, 3^ Samples making up three silvatic subpopulations selected for calculation of unbiased *F*_IS_

^4^ Samples making up one domestic subpopulation selected for calculation of unbiased *F*_IS_
