# Supplementary material for: Genome-Scale Multilocus Microsatellite Typing of Trypanosoma cruzi Discrete Typing Unit I Reveals Phylogeographic Structure and Specific Genotypes Linked to Human Infection
Source: PLoS Pathog. 2009 May 1;5(5):e1000410. doi: 10.1371/journal.ppat.1000410 (PMC2669174; doi:10.1371/journal.ppat.1000410)
Supplement: Table S3 — Microsatellite loci and primers employed in this study. (0.12 MB DOC) [file ppat.1000410.s004.doc]

**Table S3 Microsatellite loci and** primers employed in this study.

| **Contig ID^a^** | **Primer code** | **Position^b^** | **Repeat type^c^** | **Forward/Reverse Primer (5'-3')** |
| --- | --- | --- | --- | --- |
| 1047053506529 | 6529(TA)CAGT(GA) | 120977-121012 | (TA)_n_CAGT(GA)_n_ | CTAAGAAGCAGGCAACCCAG |
|  |  |  |  | CACCGCCAGCAGTAACAAAT |
| 1047053506529 | 6529(TA)a | 99171-99204 | (TA)_n_ | CGCGACACACTTCGAGATAA |
|  |  |  |  | CGCCTAGTCAAATGATGATACG |
| 1047053506529 | 6529(TCC) | 75330-75360 | (TCC)_n_ | ATTCCTTGTCGGTGAACACC |
|  |  |  |  | GCCCTCCGTTTATTCCTCTC |
| 1047053506529 | 6529(CA)a | 92287-92316 | (CA)_n_ | TGTGAAATGATTTGACCCGA |
|  |  |  |  | AGAGTCACGCCGCAAAGTAT |
| 1047053506529 | 6529(TA)b | 75669-75701 | (TA)_n_ | TGAAGGAGATTCTCTGCGGT |
|  |  |  |  | CTCTCATCTTTTGTTGTGTCCG |
| 1047053506529 | 6529(CA)b | 84365-84390 | (CA)_n_ | ACCAATACCACCACCACCAC |
|  |  |  |  | TCAGAAATCTGCTTTTCCCC |
| 1047053506559 | 6559(CAA) | 94129-94191 | (CAA)_n_ | ATTCGGCCTGTTTGTATTCG |
|  |  |  |  | TTGGTTGTGTTTTACTGCTGC |
| 1047053506559 | 6559(GA) | 16332-16526 | (GA)_n_ | GCAAAAGCAACAAAAACAGC |
|  |  |  |  | GAGCACACGAAGGGGAATAA |
| 1047053506559 | 6559(TC) | 42683..42715 | (TC)_n_ | CGCTCTCAAAGGCACCTTAC |
|  |  |  |  | ATATGGACGCGTAGGAGTGC |
| 1047053506559 | 6559(TG) | 28781-28980 | (TG)_n_ | TTGCTTGCTTTCCACGTGTA |
|  |  |  |  | TATTCCCTTTGCCTTTGCTG |
| 1047053506789 | 6789(TG) | 64001-64129 | (TG)_n_ | GAGCAGATCTTCCTTGTGCC |
|  |  |  |  | TGGTGAAATGCACGCATC |
| 1047053506855 | 6855(TA)(GA) | 5369-5576 | (TA)_n_(GA)_n_ | TGTGATCAACGCGCATAAAT |
|  |  |  |  | TTCCATTGCCTCGTTTTAGA |
| 1047053506855 | 6855(TC) | 76285-76410 | (TC)_n_ | GACATGTATGCTTGAAACCTCC |
|  |  |  |  | TCCATCTCCCTTCACACTCC |
| 1047053506925 | 6925(TG)a | 119499-119520 | (TG)_n_ | TCGTTCTCTTTACGCTTGCA |
|  |  |  |  | TAGCAGCACCAAACAAAACG |
| 1047053506925 | 6925(TG)b | 48024-48053 | (TG)_n_ | GAAACGCACTCACCCACAC |
|  |  |  |  | GGTAGCAACGCCAAACTTTC |
| 1047053506925 | 6925(CT) | 88658-88832 | (CT)_n_ | CATCAAGGAAAAACGGAGGA |
|  |  |  |  | CGGTACCACCTCAAGGAAAG |
| 1047053507093 | 7093(TAA)(A) | 39675-39924 | (TAA)_n_(A)_n_ | TCAAAGGGTGCTAATCCAAA |
|  |  |  |  | AACCATGAGGGAAGAAGAAA |
| 1047053507093 | 7093(TA)b | 51225-51373 | (TA)_n_ | GGAAACACATCACGCAAAGA |
|  |  |  |  | AGTGACAAAGGGGGACATTG |
| 1047053507093 | 7093(TC) | 69979-70016 | (TC)_n_ | CCAACATTCAACAAGGGAAA |
|  |  |  |  | GCATGAATATTGCCGGATCT |

| **Contig ID^a^** | | **Primer code** | **Position^b^** | | **Repeat type^c^** | | **Forward/Reverse Primer (5'-3')** | |  |
| --- | --- | --- | --- | --- | --- | --- | --- | --- | --- |
| 1047053507093 | 7093(TA)c | | | 16233-16257 | | (TA)_n_ | | CGTGTGCACAGGAGAGAAAA | |
|  |  | | |  | |  | | CGTTTGGAGGAGGATTGAGA | |
| 1047053507093 | 7093(TCC) | | | 25751-25779 | | (TCC)_n_ | | AGACGTTCATATTCGCAGCC | |
|  |  | | |  | |  | | AGCCACATCCACATTTCCTC | |
| 1047053508741 | 8741(TA) | | | 94443-94463 | | (TA)_n_ | | TGTAACGGTAGGTCTCAATTCG | |
|  |  | | |  | |  | | TTGCACTTGTGTATCTCGCC | |
| 1047053508741 | 8741(CT)(TA) | | | 72728-72864 | | (CT)_n_(TA)_n_ | | GCAGAGACGCACAGACACAT | |
|  |  | | |  | |  | | AAAGTGCCATCCCACCCTC | |
| 1047053510101 | 10101(CA)a | | | 88534-88549 | | (CA)_n_ | | GTCGCCATCATGTACAAACG | |
|  |  | | |  | |  | | CTGTTGGCGAATGGTCATAA | |
| 1047053510101 | 10101(TC) | | | 49478-49495 | | (TC)_n_ | | CGTACGACGTGGACACAAAC | |
|  |  | | |  | |  | | ACAAGTGGGTGAGCCAAAAG | |
| 1047053510101 | 10101(CA)b | | | 15729-15742 | | (CA)_n_ | | ACCCAGAGGGGAGAAAAAGA | |
|  |  | | |  | |  | | TTTACGGTTGGTTCGTGTGA | |
| 1047053510101 | 10101(TAA)b | | | 987-1016 | | (TAA)_n_ | | CCGCGGTAGAAGAACCATAA | |
|  |  | | |  | |  | | TGCGTATTCACGACGAGAAG | |
| 1047053510101 | 10101(TA) | | | 46598-46638 | | (TA)_n_ | | AACCCGCGCAGATACATTAG | |
|  |  | | |  | |  | | TTCATTTGCAGCAACACACA | |
| 1047053510101 | 10101(CA)c | | | 87584-87606 | | (CA)_n_ | | GTGTCGTTGCTCCCAAACTC | |
|  |  | | |  | |  | | AAACTTGCCAAATGTGAGGG | |
| 1047053510187 | 10187(TG)a | | | 124985-125219 | | (TG)_n_ | | GCGCGTTATTAACACTCGCT | |
|  |  | | |  | |  | | GCCCGGTATCATTGAAAAGA | |
| 1047053510187 | 10187(CA) | | | 83338-83417 | | (CA)_n_ | | CTACCTTCTCTTTCCTCCCTAACC | |
|  |  | | |  | |  | | TTTGCTCTGGACTGCATGC | |
| 1047053510187 | 10187(TG)b | | | 63326-63566 | | (TG)_n_ | | AAGAGAGGCACTCCCTGTGA | |
|  |  | | |  | |  | | GAGGAAGAGGAAGTACAGTTGAGC | |
| 1047053510187 | 10187(TA) | | | 44002-44057 | | (TA)_n_ | | AGAAAAAGGTTTACAACGAGCG | |
|  |  | | |  | |  | | CGATGGAGAACGTGAAACAA | |
| 1047053510187 | 10187(GA) | | | 71097-71226 | | (GA)_n_ | | GTCACACCACTAGCGATGACA | |
|  |  | | |  | |  | | ACTGCACAATACCCCCTTTG | |
| 1047053510187 | 10187(TTA) | | | 32430-32629 | | (TTA)_n_ | | GAGAGAGATTCGGAAACTAATAGC | |
|  |  | | |  | |  | | CATGTCCCTTCCTCCGTAAA | |
| 1047053510187 | 10187(CA)(TA) | | | 39618-39874 | | (CA)_n_(TA)_n_ | | CATGTCATTAAGTGGCCACG | |
|  |  | | |  | |  | | GCACATGTTGGTTGTTGGAA | |
| 1047053510359 | 10359(TTG) | | | 24890-25024 | | (TTG)_n_ | | ATGGGTGCGAGAGGTATGTC | |
|  |  | | |  | |  | | TGTCAAAACAGCGGAAAGAA | |
| 1047053511071 | 11071(CA)(GA) | | | 1001-10154 | | (CA)_n_(GA)_n_ | | ACGACCAAAGCCATCATT | |
|  |  | | |  | |  | | GATGCTAACTGCTCAAGTGA | |

| **Contig ID^a^** | **Primer code** | **Position^b^** | **Repeat type^c^** | **Forward/Reverse Primer (5'-3')** |
| --- | --- | --- | --- | --- |
| 1047053511283 | 11283(TG) | 89251-89397 | (TG)_n_ | TGCTCTTGTTGTTGGAGTGC |
|  |  |  |  | CAGCAGACAATCCAACCAAC |
| 1047053511283 | 11283(A)(CA) | 58996-59147 | (A)_n_(CA)_n_ | GCGGTACACCAACATGTACG |
|  |  |  |  | GTGTGTTTGTGTGTGAGAGGC |
| 1047053511283 | 11283(TCG) | 88680-88708 | (TCG)_n_ | ACCACCAGGAGGACATGAAG |
|  |  |  |  | TGTACACGGAACAGCGAAG |
| 1047053511283 | 11283(TA)b | 12639-12819 | (TA)_n_ | AACATCCTCCACCTCACAGG |
|  |  |  |  | TTTGAATGCGAGGTGGTACA |
| 1047053511863 | K638* | 7458-7500 | (CA)_n_ | AGTTGACATCCCCAAGCAAG |
|  |  |  |  | CCCTGATGCTGCAGACTCTT |
| Unknown | TcUn1 | Unknown | Unknown | TGAGAGTGAGGGGAGAGAGC |
|  |  |  |  | GGGCACATTATGTGTGTGCT |
| Unknown | TcUn2 | Unknown | Unknown | AACAAAATCTAGCGTCTACCATCC |
|  |  |  |  | GGTGTTGGCGTGTATGATTG |
| Unknown | mclf10** | Unknown | (CA)_n_A(CA)_n_ | GCGTAGCGATTCATTTCC |
|  |  |  |  | ATCCGCTACCACTATCCAC |
| Unknown | scle10** | Unknown | (CT)_n_(TG)_n_ | GATCCCGCAATAGGAAAC |
|  |  |  |  | GTGCATGTTCCATGGCTT |
| Unknown | TcUn3 | Unknown | Unknown | CTTAAAGAGATACAAGAGGGAAGG |
|  |  |  |  | CTGTTATTTCAATAACACGGGG |

^a^ Refers to a sequence fragment identified by the *T. cruzi* genome project (www.**tigr**.org).

^b^ Refers to the position of the of the primer binding site in the flanking region 5’-3’ along the sense strand. Unidentified positions are marked ‘unknown’.

^c^ Refers to the repeat type (e.g. di-nucleotide, tri-nucleotide). Unidentified repeat types are marked ‘unknown’.

* K638 published in Gaunt *et al.,* (2003) [6]

** mclf10 and scle10 published in Oliveira *et al*., (1998) [15]
